# History and Perspectives of Nuclear Medicine in Bangladesh

**DOI:** 10.7508/aojnmb.2016.04.009

**Published:** 2016

**Authors:** Raihan Hussain

**Affiliations:** National Institute of Nuclear Medicine and Allied Sciences, Dhaka, Bangladesh

**Keywords:** Bangladesh, History, Nuclear Medicine

## Abstract

Bangladesh is one of the smaller states in Asia. But it has a long and rich history of nuclear medicine for over sixty years. The progress in science and technology is always challenging in a developing country. In 1958, work for the first Nuclear Medicine facility was commenced in Dhaka in a tin-shed known as ‘Radioisotope Centre’ and was officially inaugurated in 1962. Since the late 50s of the last century nuclear medicine in Bangladesh has significantly progressed through the years in its course of development, but still the facilities are inadequate. At present there are 20 nuclear medicine establishments with 3 PET-CTs, 42 gamma camera/SPECTs with 95 physicians, 20 physicists, 10 radiochemists and 150 technologists. The Society of Nuclear Medicine, Bangladesh (SNMB) was formed in 1993 and publishing its official journal since 1997. Bangladesh also has close relationships with many international organizations like IAEA, ARCCNM, AOFNMB, ASNM, WFNMB and WARMTH. The history and the present scenario of the status of nuclear medicine in Bangladesh are being described here.

## Introduction

**Figure 1:**
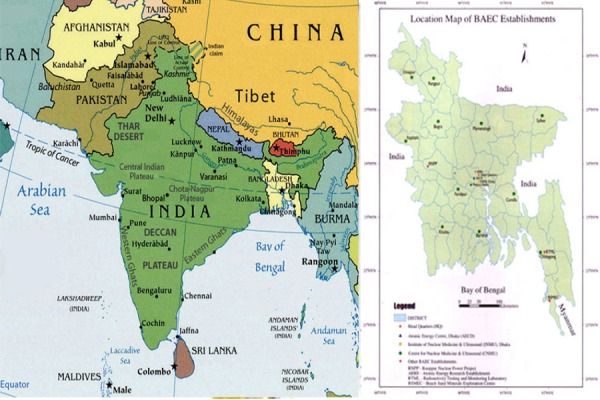
a. Location of Bangladesh in map of Asia, b. Locations of major Nuclear Medicine establishments in Bangladesh

Bangladesh is a small country of only 147,570 sq km in the eastern part of the Indian sub-continent (Figure 1). It lies between India and Myanmar and geographically it is mainly a flat alluvial plain in the gangetic delta. Traditionally it is based on agricultural economy. It has the highest population density in the world (160 million, 1084/km2) with its expected challenges. Moreover the country is prone to natural calamities, like, flood, cyclones, tidal bore and droughts. Nevertheless the country is progressing fast with annual GDP growth of 6-7% for the last few years suggesting determination of the people. Similarly nuclear medicine has also been growing for the last decades and we will go back the timeline of the development and progress of Nuclear Medicine (NM) in this article.

## History

**Figure 2:**
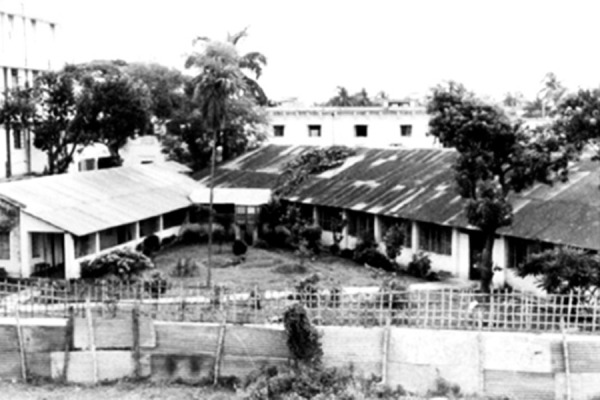
The first Nuclear Medicine establishment of the country “Radioisotope Centre” in Dhaka

The history of NM in Bangladesh is quite old. More than 50 years have passed since the first NM centre started its journey (1). Plans were made in late 1950s under the Pakistan Atomic Energy Commission (PAEC) for establishment of the first NM department in the Dhaka Medical College, the only medical college in the then East Pakistan. The construction started in 1958 in a tin-shed known as ‘Radioisotope Centre’ and Dr. Mazharul Islam joined there as a medical officer (Figure 2). It was officially inaugurated in 1962 by Gen. Barki, the then Health Minister of Pakistan. But the department lacked regular supply of isotopes and had a rectilinear scan, two probe renograms and a radioactive iodine uptake system. It faced some administrative problems due to dual administration (both by Ministry of Health as well PAEC). So no significant up-gradation was done. In early 1960’s PAEC took initiative to develop NM in the region. At that time Dr. Kamaluddin Ahmed joined PAEC. He went to West Pakistan in Karachi to work and later went to UK for higher studies in 1962 and achieved Masters of Sciences (MSc.). He returned back to East Pakistan in 1966 and contributed immensely in development of NM. Actually his determination and hard work lead to the growth of NM in the then East Pakistan and later on in Bangladesh. So he is considered as the Father of NM in the country.

In 1960’s, two more centres were constructed, one in the south-east part in Chittagong and another in the west in Rajshahi by PAEC. In the mid 60’s Dr. Shawkat Jahan and Dr. Kamaruddin Pramanik also joined NM in Dhaka and Lahore respectively. Dr. Kamaluddin Ahmed on his return from UK joined in Dhaka NM centre in 1966. He could motivate the authorities for improvement of the facilities and moved to a newly established centre in Chittagong. Dr. Kamaruddin Pramanik joined in Rajshahi from Lahore in 1970. By this time there were three NM facilities in the country. Dr. Pramanik is known to have revolutionary ideas with new techniques, as for example introduction of radioisotopic gold needle per rectum for rectal cancer.

**Figure 3:**
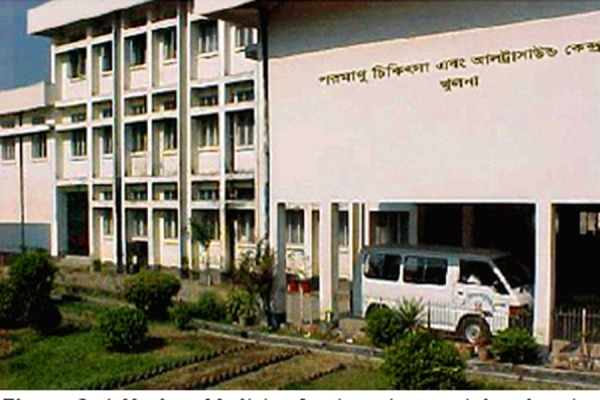
A Nuclear Medicine Institute in a peripheral region of the country

In 1971 war of independence took place. In a war ravaged country NM suffered difficulties as in any other sectors. After the independence Bangladesh Atomic Energy Commission (BAEC) was founded in February 1973 and steps were taken for promotion of NM (2). Two new centres, one in the north-west in Dinajpur and another in the north-east in Sylhet were established in 1980. It may be mentioned that the northern part of the country was known for endemic iodine deficiency zone. By creating these centres awareness about NM among the medical community started to grow. For the general public, these centres proved to be essential as these catered for thyroid diseases. In 1980 the Radioisotope Centre in Dhaka was handed over to BAEC by the ministry of health for better management. By 1983, another four centres in Mymensingh, Rangpur, Barisal and Khulna were constructed. In 1997, 4 new centres were constructed at Faridpur, Comilla, Bogra and Mitford Hospital, Dhaka under BAEC (Figure 3). In the recent years private sectors joined in expansion of Nuclear Medicine and 5 centres were setup. BAEC has very recently established another centre at Cox’ Bazar. Apart from BAEC, different governmental bodies have also established 3 more centres.

## Education

In early 1980s a centralised institute to look after the human resources development and NM education was felt. The Institute of Nuclear Medicine (INM) was established in the Institute of Postgraduate Medicine and Research (IPGMR) campus located in central Dhaka city in 1980. It started functioning in 1981 and marked as a milestone in the development of NM in the country. First ultrasound machine and computerised gamma camera of the country was established in 1981 and 1983 respectively.

The then INM became an affiliated institute of Dhaka University and course curriculum for one year diploma course was approved in 1987. In 1988 the first batch of Diploma in Nuclear Medicine (DNM) were enrolled in Dhaka University. This was another great leap forward. This created an opportunity for the local young professionals for higher education. Gradually candidates from regional countries like Nepal also got enrolled and completed the course. Eventually after ten years, the one year course was extended to two years masters program (Master of Philosophy) which is still continuing. Now the course is being planned and to be extended for four years (Residency program).

## Current situation

Steps are taken to establish more centres in near future (4 under BAEC, 2 under ministry of health, 4 under private sectors). So it can be easily understood that NM is an expanding arena in the country. Regarding the equipment, the first single headed SPECT was installed in 1994, first double headed SPECT in 2003, First SPECT-CT in 2008 and first PET-CT in 2010. At present there are 20 NM centres with 3 PET-CTs, 42 gamma camera/SPECTs having 95 NM physicians, 20 physicists, 10 radiochemists and 150 technologists (3). The National Institute of Nuclear medicine and Allied Sciences (NINMAS) in Dhaka is by far the largest NM facility in the country. It covers an area of 60,000 sq. ft. and has seven working divisions, namely, Scintigraphy, Nuclear Cardiology, Nuclear Nephrology, In-vitro, Thyroid, Ultrasound and Colour Doppler and R & D divisions. According to the departmental statistics, the number of patients catered at NINMAS was 54678, 60569, 62192 and 63377 in the fiscal years of 2010-11, 2011-12, 2012-13 and 2013-14 respectively (4). Bangladesh also organizes various NM training programs and workshops, in which both local and foreign participants take part. International Atomic Energy Agency (IAEA) is also providing placement of foreign trainees to Bangladesh for Fellowship training.

## Society of Nuclear Medicine, Bangladesh (SNMB)

The Society of Nuclear Medicine, Bangladesh (SNMB) was formed in 1993 with Dr. Kamaluddin Ahmed as the founder president and Dr. M A Karim as the founder general secretary. The first national conference was held in 1994 at the Atomic Energy Centre (AEC) Auditorium in Dhaka. SNMB was established for promotion and development of activities of Nuclear Medicine and allied sciences in Bangladesh.

**Figure 4:**
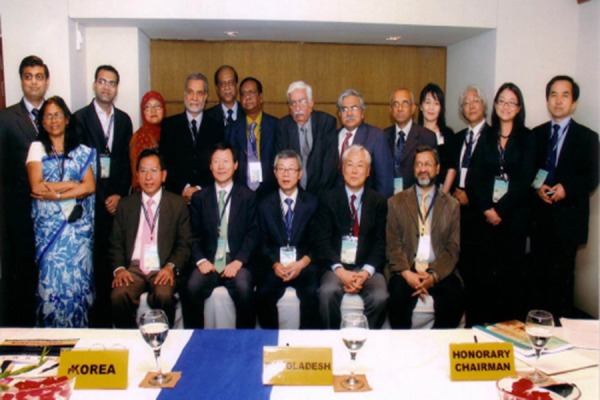
A group of Nuclear Medicine personalities of Asian region at the ARCCNM meeting in Dhaka, Bangladesh, 2010

**Figure 5:**
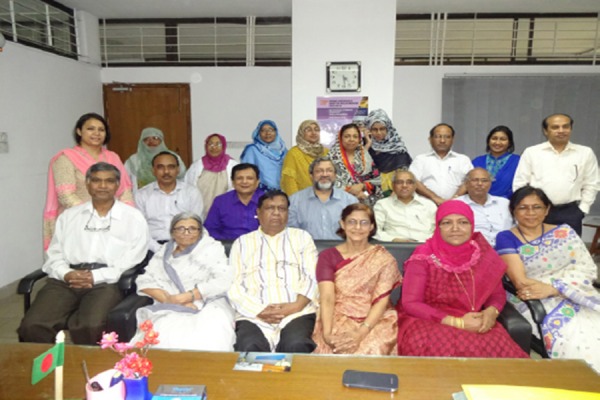
Some of the senior members of Society of Nuclear Medicine (SNMB)

SNMB functions actively for the promotion and consolidation of the subject. Annually it holds conferences and seminars to enrich the discipline among the members. It is also publishing “The Bangladesh Journal of Nuclear Medicine (BJNM)” regularly for the last 18 years (http://banglajol.info/index.php/BJNM). SNMB has also successfully organised international NM events like Asian Regional Cooperative Council for Nuclear Medicine (ARCCNM) conferences in 2003 and 2010 (Figure 4). The society has now more than 100 members and considered one of the vibrant and active medical societies in the country (Figure 5). It is also known to be one of the most active NM societies in the south-east Asian region. Two of our senior members Prof. MA Karim and Prof. Shahana Afroz have attained the post of Chairman of BAEC, a very important position being from NM background as anywhere in the world.

SNMB held its 20^th^ national conference very recently on 20-22 March, 2015. It was a grand occasion of Nuclear Medicine (NM) in Bangladesh and was attended by more than 150 participants from all over the country as well as very prominent international leaders like Prof. Henry Bom, President AOFNMB and Prof. Jun Hatazawa, Chairman ARCCNM. At present SNMB stands on a solid foundation with one hundred members.

## International Activities

SNMB actively cooperates with Asia-Oceania Federation of Nuclear Medicine and Biology (AOFNMB), ARCCNM and Asian School of Nuclear Medicine (ASNM), who are working together for the excellence of NM scenario in all aspects of scientific and academic activities, human resources development and promotion of newer technologies in the region (5). SNMB also has close relationship with global bodies like World Federation of Nuclear Medicine and Biology (WFNMB) and World Association of Radiopharmaceutical & Molecular Therapy (WARMTH). The members of the society also actively participate in various NM conferences throughout the world and also take active roles in different organisational posts. At present I am serving as Secretary General of AOFNMB and Treasurer of ARCCNM. I was also an elected Member of the Governing Body of WARMTH in 2012 - 2015. Dr. Mizanul Hasan is the Vice Dean of ASNM. Dr. Kamaluddin Ahmed and Dr. M A Karim have worked as IAEA experts in promoting NM in African and Asian countries. These are some examples of our member’s international collaborations.

## Conclusion

NM in Bangladesh has significantly progressed through the years in its course of development by the government initiatives as well as cooperation from IAEA and above all by the dedication and efforts of the NM personals. The country has also maintained international relationship with ARCCNM, AOFNMB, ASNM, WFNMB and WARMTH for coordinated approach of development. The facilities are still inadequate as being a developing country and a long way to go especially in a land of 160 million people.
